# Association between dietary inflammatory index and oral cancer risk: A systematic review and dose–response meta-analysis

**DOI:** 10.3389/fonc.2022.920452

**Published:** 2022-09-26

**Authors:** Zhicheng Luo, Xidi Zhu, Yingyun Hu, Shipeng Yan, Lizhang Chen

**Affiliations:** ^1^ Department of Cancer Prevention and Control, Hunan Cancer Hospital/the Affiliated Cancer Hospital of Xiangya School of Medicine, Central South University, Changsha, China; ^2^ Department of Epidemiology and Health Statistics, Xiangya School of Public Health, Central South University, Changsha, China

**Keywords:** oral cancer, dietary inflammatory index, diet, meta-analysis, dose-response relationship

## Abstract

**Background:**

Dietary inflammatory index (DII) has been suggested to be associated with oral cancer risk. However, a quantitative comprehensive assessment of the dose–response relationship has not been reported. We performed a meta-analysis to clarify the risk of oral cancer with DII.

**Methods:**

We searched PubMed, Embase, Cochrane Library, and Web of Science databases for relevant articles published up to 1 March 2022. Fixed- or random-effects models were utilized to estimate the pooled odds ratio (OR) of oral cancer with DII, as appropriate. Restricted cubic splines were used to model the dose–response relationship.

**Results:**

We included five case–control studies involving 1,278 cases and 5,137 controls in the meta-analysis. Risk of oral cancer was increased by 135% with the highest versus lowest DII level [OR: 2.35, 95% confidence interval (CI): 1.88–2.94], and 79% with higher versus lower DII level (OR: 1.79, 95% CI: 1.49–2.15). We found no evidence of a nonlinear dose–response association of DII with oral cancer (*p_non-linearity_* = 0.752), and the risk was increased by 17% (OR: 1.17, 95% CI: 1.05–1.30) with 1 unit increment in DII score.

**Conclusion:**

This meta-analysis suggested that a higher DII score was associated with increased risk of oral cancer. Therefore, reducing pro-inflammatory components and promoting anti-inflammatory components of diet may be effective in the prevention of oral cancer.

## Introduction

Oral cancer is the most prevalent subtype of head and neck cancers, and has become a major public health problem. It has long occupied one of the top 20 leading causes of morbidity and mortality worldwide (1, 2). According to the latest Global Cancer Observatory report, there were an estimated 377,713 new cases and 177,757 deaths of lip and oral cavity globally in 2020 (2). Tobacco, alcohol, and betel quid consumption have been identified as the major causes of oral cancer (3). In addition, accumulated epidemiological evidence suggested that diet also played a critical role in the occurrence of oral cancer (4–6). Inflammation and oxidative stress triggered by certain dietary components may be a potential mechanism. For example, red meat and sugar-sweetened beverage with strong pro-inflammatory effects have been shown to be associated with cancer in numerous observational and experimental studies (7–10).

To more comprehensively and scientifically represent the inflammatory potential of diet, Shipappa et al. developed the dietary inflammatory index (DII), which was derived from a literature review of 1,943 articles (11). The original DII includes a total of 45 food components, each with an estimated inflammatory potential. To be specific, one of three points was assigned to each food parameter based on whether they (1) had significantly increased (+1), decreased (−1), or insignificant (0) effect on the four established pro-inflammatory biomarkers: interleukin-1beta (IL-1β), interleukin-6 (IL-6), tumor necrosis factor-alpha (TNF-α), and C-reactive protein (CRP); or (2) had significantly decreased (+1), increased (−1), or insignificant (0) effect on the two established anti-inflammatory biomarkers: interleukin-4 (IL-4) and interleukin-10 (IL-10). The point was first weighted by study characteristics including types of study and study designs. An overall inflammatory effect score for each food parameter was then calculated by dividing the weighted pro- and anti-inflammatory articles by the total weighted number of articles, followed by subtracting the anti- from the pro-inflammatory fraction. The raw overall score was weighted again according to a cutoff point of 236, which was the median of the total weighted number of articles for all food parameters. The full value of the inflammatory effect score was retained if the total number of weighted articles for a particular food parameter was not less than 236; otherwise, a weighted ratio of the total number of weighted articles to 236 was multiplied by the raw inflammatory effect score. Depending on whether this score was greater than 0, 9 food components, namely, energy, carbohydrate, cholesterol, total fat, saturated fat, trans fat, protein, iron, and vitamin B_12_, have pro-inflammatory features (>0), and another 36 food components have anti-inflammatory properties (<0). In terms of dietary intake data, it was linked to the regionally representative world database to obtain a *Z*-score and corresponding centered percentile value for each food parameter. Finally, the centered percentile of each food parameter was multiplied by its respective inflammatory effect score, and then all 45 scores were summed to create an overall DII score for each participant. Sometimes, the DII score was further adjusted for total energy intake to control for its potential effects. In practice, the total DII score has been proven to be positively correlated with the concentrations of several common inflammatory biomarkers, such as CRP, interleukin-2 (IL-2), and TNF-α (12, 13). In summary, DII provided a new perspective to investigate the relationship between diet-related inflammation and diseases.

In recent years, DII has been implicated in the occurrence and development of several types of cancer, such as breast cancer, colorectal cancer, and gastric cancer (14–17). There were also existing literature examining the relationship between DII and oral cancer (18–22). However, the strength of the correlation varies among studies. In addition, although two systematic reviews and meta-analyses have been published in 2019 for DII and upper aerodigestive tract cancers, oral cancer as a subtype was not independently analyzed due to insufficient relevant studies (23, 24). Therefore, we aimed to comprehensively assess the dose–response relationship between DII and oral cancer risk based on a systematic review and meta-analysis updated to March 2022. To the best of our knowledge, this is the first meta-analysis to quantify the dose–response relationship.

## Methods

### Protocol

The systematic review and meta-analysis was conducted according to the Preferred Reporting Items for Systematic Reviews and Meta-Analyses (PRISMA) (25). To define the inclusion criteria, the PICOS framework was described as follows:

In the form of PICOS (population, intervention/exposure, comparator, outcome, and study design), the study was described as follows:

Population: not specified;

Exposure: participants at the highest or higher DII level;

Comparator: participants at the lowest or lower level of DII level;

Outcome: occurrence of oral cancer;

Study design: observational studies including prospective cohort, case–control, or cross-sectional studies.

### Literature search strategy

We systematically searched PubMed, Embase, Cochrane Library, and Web of Knowledge database for literatures reporting the association of DII with oral cancer that were published up to 1 March 2022. The search strategy was designed by combining the following MeSH terms with free words: dietary inflammatory index; dietary inflammatory score; dietary score; inflammatory diet; inflammatory potential of diet; dietary inflammation potential; inflammatory potential intake; anti-inflammatory diet; pro-inflammatory diet; dietary pattern; diet-related inflammation; index-based dietary patterns; DII; mouth neoplasms; mouth neoplasm; oral neoplasms; oral neoplasm; mouth cancers; mouth cancer; oral cancers; oral cancer; cancer of mouth; cancer of the mouth; oral squamous cell carcinoma; oral squamous cell cancer. The details of the search strategy are shown in **Supplementary Table 1**. We also searched the reference lists of review publications and identified publications for more relevant studies.

### Study selection

Studies were included if they (1) were prospective cohort, case–control, or cross-sectional studies; (2) investigated the association between DII and risk of oral cancer; (3) reported estimates of the hazard ratio (HR), relative risk (RR), or odds ratio (OR) with their 95% confidence intervals (Cis) or sufficient information to calculate these; and (4) provided quantitative data of DII as well as number of cases and participants, or exposed person-years for the dose–response analysis.

### Data extraction

Two investigators (Luo and Zhu) independently extracted data using a designed extraction form, and any disagreements were resolved by discussion. The collected information was as follows: name of the first author, publication year, country where the study was conducted, study design, study duration, participants characteristics (sex and age), DII assessment and measures, DII ranges or median or mean for each category, risk estimates with corresponding 95% CIs of oral cancer, and covariates adjusted in each included study.

### Study quality assessment

The Newcastle–Ottawa scale (NOS) was used to assess the quality of the included studies by two authors (Luo and Zhu) (26). The scale contains nine questions with each answer scoring 0 or 1 point. Thus, the total score was up to 9 points and 7–9 points were considered to represent high quality.

### Statistical methods

In this meta-analysis, the term “odds ratio (OR)” was regarded as the unified effect size for all studies (27). To estimate the pooled ORs for the association between DII and oral cancer risk, we used fixed- or random-effects models depending on the presence of statistically significant heterogeneity. Three series of forest plots were generated for DII of (1) the highest category vs. the lowest category; (2) higher category vs. lower category; and (3) a 1-unit increment. For analysis of the highest DII in comparison with the lowest DII, we extracted the ORs of oral cancer at the highest DII category in each original article. That is to say, the third, fourth, and fifth DII category were aimed when grouped by tertile, quartile, and quintile of DII score, respectively. For analysis of higher DII in comparison with lower DII, the reference category was defined as lower DII, while the other categories in the same study were pooled into a new group as higher DII (28). For example, if an original article classified participants according to the quartiles of DII score, we synthesized the three ORs associated with the second, third, and fourth quarter DII levels into a single OR as higher DII. For analysis of continuous DII, we directly extracted the OR associated with a 1-unit increment in DII score if the value was provided by the original article; or we would calculate the value by using the method of Greenland and Longnecker (29) when continuous DII from the original article was not available.

We also investigated possible linear or non-linear associations of DII with oral cancer risk by using the method of Greenland and Longnecker (29). This method was only for those studies with at least three quantitative DII categories, and each category contained the corresponding sample size, number of cases, dose value of DII, and OR with 95% CI. If the mean or median value of DII in any category was not directly provided, the midpoint of lower and upper boundaries in each category was employed as the mean or median (30). If the lowest or highest category was open-ended, the DII value was calculated as the endpoint value for that category plus or minus the dose interval value for the adjacent category (31). The dose–response relationship between DII and oral cancer risk was examined by using restricted cubic splines (RCS) with 3 knots at fixed percentiles (25th, 50th, and 75th) of the distribution of DII values (27).

Heterogeneity of effect size was evaluated by inconsistency index (*I* ^2^) and Cochran *Q* test (32). *I* ^2^ value > 50% or *p*-value < 0.05 was considered statistically significant. We performed subgroup analyses to explore potential sources of heterogeneity, including geographic area, years of study duration, study quality, NOS score, number of cases, number of DII components, energy-adjusted DII (E-DII), and the covariates adjusted in the analysis [age/gender, socio-economic status (SES), body mass index (BMI), and family history of cancer]. For analysis, we considered SES adjustment as adjusting for one or more of the following factors: occupation, income level, and educational level. In addition, sensitivity analysis was conducted to examine the potential impact of each study on pooled risk estimations through omitting one study at a time. Potential publication bias was evaluated by the Begg’s and Egger’s tests, and *p*-value at < 0.05 was considered to indicate publication bias (33, 34). All analyses were performed by Stata 14.0 (Stata Corp, College Station, TX, USA). All tests were two-sided, with *p* < 0.05 considered statistically significant.

## Results

**Figure 1:**
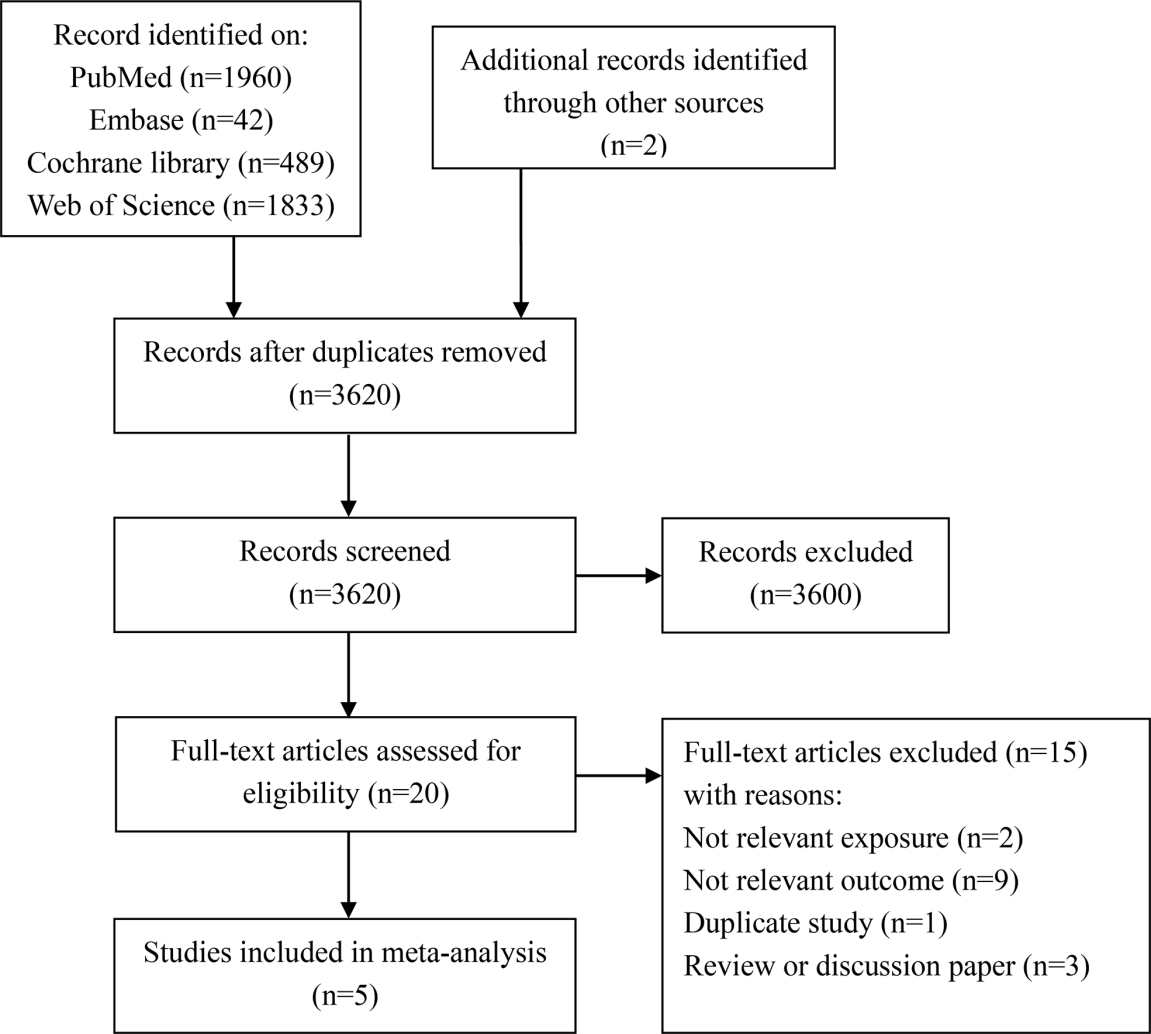
Flowchart of literature search and study selection for systematic review and meta-analysis.

We initially identified 4,324 articles from PubMed, Embase, Cochrane Library, and Web of Science databases and two from other sources. After excluding duplicates (*n* = 706) and evaluating the abstracts and titles of the articles (*n* = 3,600), we obtained 20 full-text articles. Next, we further excluded 15 articles for the following reasons: irrelevant exposure or outcome (*n* = 11), duplicate study (*n* = 1), and review or discussion paper (*n* = 3). Finally, five studies were included in this meta-analysis. The detailed literature screening process is shown in **Figure 1**.

### Characteristics and quality assessment of included studies

The included five studies were all case–control studies with a total of 1,278 cases and 5,137 controls. Among these studies, two were from Asia, two were from America, and one was from Europe (**Table 1**). All articles applied food-frequency questionnaires (FFQ) to collect food intake information. Then, DII was calculated based on FFQ using the method of Shivappa et al. Four of five studies used E-DII as the exposure. **Supplementary Table 2** shows the food parameters included in DII and covariates adjusted in each study. All studies reported ORs of oral cancer with categorical DII, and only three studies reported continuous DII. Assessment of quality suggested that two of five articles were of high quality (NOS ≥ 7). **Supplementary Table 3** provides the detailed NOS scoring information for each included study.

**Table 1:** General characteristics of included studies in the meta-analysis of DII and oral cancer risk.

Study	Races/nationalities	Study design	Sample size(case/control)	Mean/median age(case/control)	Male/female(case/control)	DII measurement (no. of DII components)	E-DII	Years of duration	Study quality(NOS score)
Bao et al., 2020 (18)	Chinese	Matched case–control	295/425	57.80/59.22	185/295 110/130	159-item FFQ (22)	Yes	8	6
Secchi et al., 2019 (19)	Argentinean	Matched case–control	27/86	Not reported	15/46 12/40	127-item FFQ (25)	Yes	3	6
Mazul et al., 2018 (20)	American	Matched case–control	195/1,372	Not reported	966/945 302/427	72-item FFQ (27)	Yes	4	7
Abe et al., 2018 (21)	Japanese	Matched case–control	255/762	60/60	826/2,466 202/615	47-item FFQ (22)	No	4.8	6
Shivappa et al., 2017 (22)	Italian	Matched case–control	506/2,492	58/58	756/1,497 190/995	78-item FFQ (31)	Yes	17	7

DII, dietary inflammatory index; FFQ, food frequency questionnaires; E-DII, energy adjusted DII; NOS, Newcastle–Ottawa Quality Assessment Scale.

### Risk of oral cancer with categorical DII

**Figure 2:**
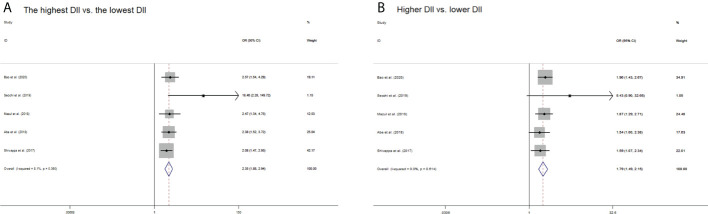
Forest plots of pooled odds ratios (ORs) with corresponding 95% confidence intervals (CIs) of oral cancer risk for **(A)** the highest DII versus the lowest DII and **(B)** higher DII versus lower DII.

When comparing the highest versus the lowest DII level, the pooled OR of oral cancer was 2.35 (95% CI: 1.88–2.94), with low heterogeneity (*I* ^2^ = 8.1%, *p_heterogeneity_* = 0.360) (**Figure 2A**). When comparing the higher versus the lower DII level, the pooled OR of oral cancer was 1.79 (95% CI: 1.49–2.15), with extremely low heterogeneity (*I* ^2^ = 0.0%, *p_heterogeneity_* = 0.614) (**Figure 2B**).

### Dose–response association between DII and oral cancer

**Figure 3:**
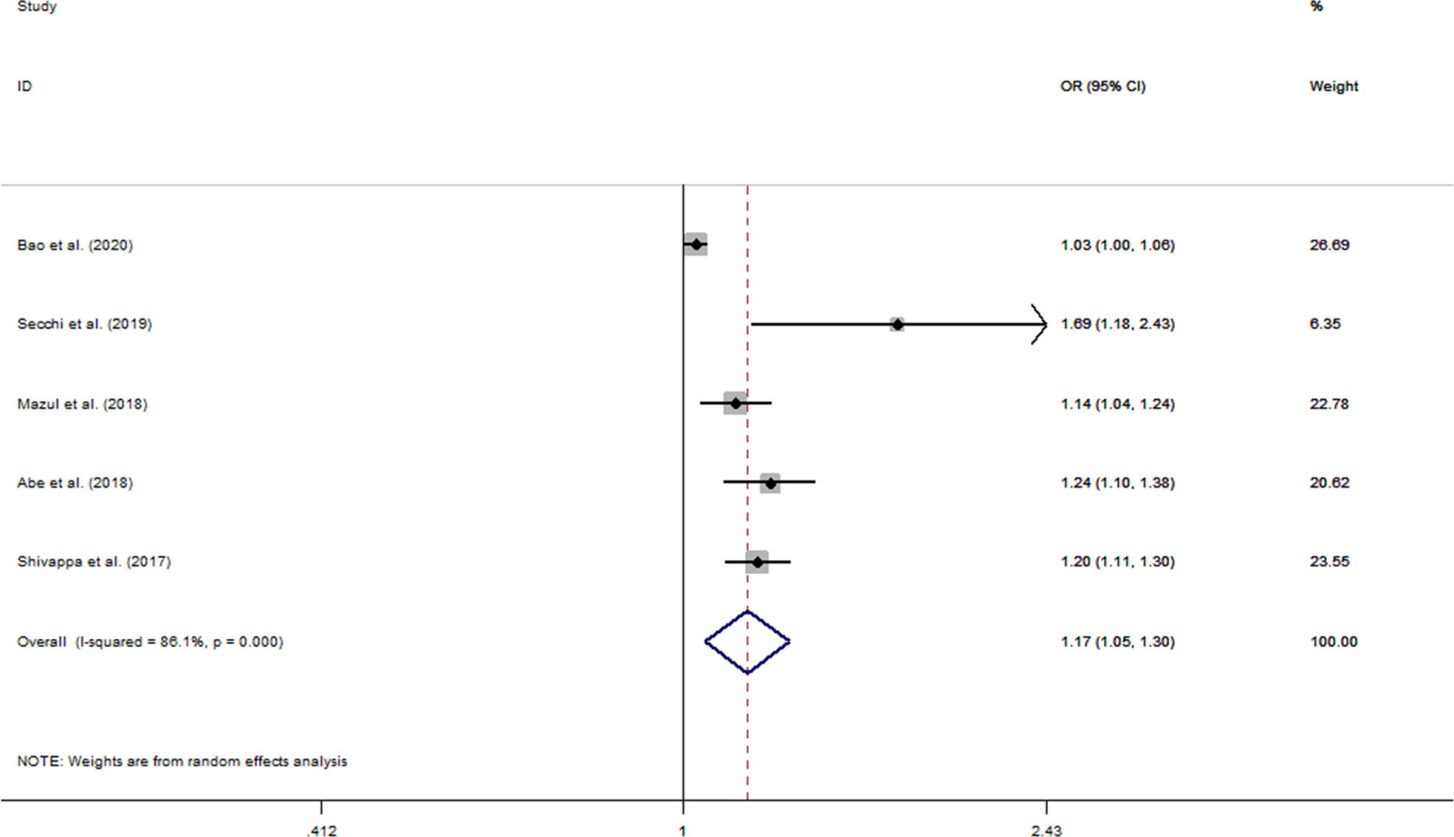
Forest plots of pooled odds ratios (ORs) with corresponding 95% confidence intervals (CIs) of oral cancer risk for per 1-unit increase in DII score.

**Figure 4:**
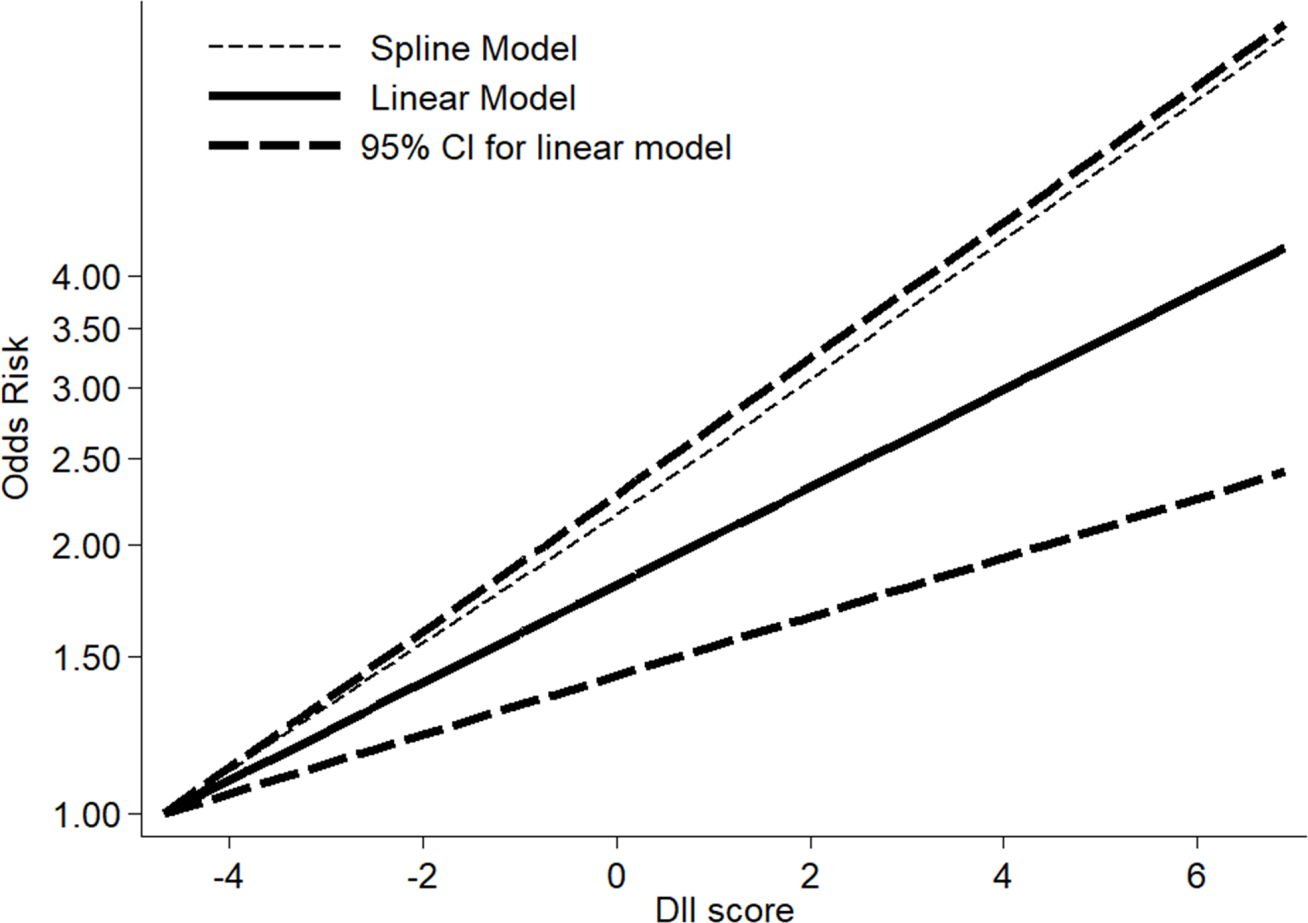
Linear and non-linear dose–response relationship between DII and risk of oral cancer.

The pooled OR for a 1-unit increment in DII was 1.17 (95% CI: 1.05–1.30) (**Figure 3**). However, significant heterogeneity was observed among studies included (*I* ^2^ = 86.1%, *p_heterogeneity_* < 0.001). We applied RCS with three knots to analyze the dose–response relationship between elevated DII and oral cancer risk (**Figure 4**). The results showed no evidence of a nonlinear association of DII with oral cancer (*p_non-linearity_* = 0.752), and meanwhile indicated that risk of oral cancer increased linearly along with increments in DII score (*p_linearity_* < 0.001).

### Subgroup and sensitivity analyses

We performed subgroup analyses to explore the sources of heterogeneity stratified by geographic area, study duration, study quality, NOS score, number of cases, number of DII components, E-DII, and adjustment for confounding factors (age/gender, SES, BMI, and family history of cancer). For the highest DII versus the lowest DII and higher DII versus lower DII, the associations between DII and oral cancer risk were consistent across strata of all the analyzed factors (all *p_heterogeneity among subgroups_* > 0.05) (**Table 2**). However, the risk of oral cancer for a 1-unit increment in DII appeared to be more pronounced in those unadjusted subgroups by SES (1.69 vs. 1.14, *p_heterogeneity among subgroups_* = 0.039) and family history of cancer (1.19 vs. 1.03, *p_heterogeneity among subgroups_* < 0.001). With sensitivity analysis omitting one study at each time, none of the individual studies had a substantial change on the pooled results (**Supplementary Figure 1**). According to Cochrane Collaboration, publication bias should be tested only when there are at least 10 studies included in the meta-analysis (35). Therefore, we did not perform Begg’s or Egger’s tests for publication bias because our meta-analysis only included five studies.

**Table 2:** Subgroup analysis results for DII and oral cancer risk.

Subgroups	No. of studies	The highest DII vs. the lowest DII				Higher DII vs. lower DII				DII, per 1 unit			
		Pooled OR (95% CI)	*I* ^2^ (%)	*p^a^*	*p^b^*	Pooled OR (95% CI)	*I* ^2^ (%)	*p^a^*	*p^b^*	Pooled OR (95% CI)	*I* ^2^ (%)	*p^a^*	*p^b^*
All studies	5	2.35 (1.88, 2.94)	8.1	0.360	–	1.79 (1.49, 2.15)	0.0	0.614	–	1.17 (1.05, 1.30)	86.1	<0.001	–
Region					0.231				0.594				0.174
Asia	2	2.46 (1.76, 3.45)	0.0	0.825		1.81 (1.40, 2.33)	0.0	0.380		1.12 (0.94, 1.34)	89.3	0.002	
Europe	1	2.08 (1.47, 2.93)	–	–		1.59 (1.07, 2.34)	–	–		1.20 (1.11, 1.30)	–	–	
North America	1	2.47 (1.34, 4.75)	–	–		1.87 (1.29, 2.71)	–	–		1.14 (1.04, 1.24)	–	–	
South America	1	18.46 (2.28, 149.72)	–	–		5.43 (0.90, 32.65)	–	–		1.69 (1.18, 2.43)	–	–	
Years of duration					0.545				0.924				0.301
≤5	3	2.56 (1.79, 3.67)	43.5	0.170		1.77 (1.34, 2.35)	0.0	0.373		1.23 (1.08, 1.40)	60.4	0.080	
>5	2	2.22 (1.67, 2.96)	0.0	0.502		1.81 (1.42, 2.30)	0.0	0.408		1.11 (0.95, 1.29)	92.1	<0.001	
Study quality					0.355				0.729				0.782
NOS score, ≤7	3	2.59 (1.86, 3.61)	43.2	0.172		1.85 (1.44, 2.37)	8.6	0.335		1.21 (0.99, 1.47)	87.7	<0.001	
NOS score, >7	2	2.16 (1.60, 2.93)	0.0	0.640		1.73 (1.32, 2.26)	0.0	0.549		1.17 (1.10, 1.24)	0.0	0.378	
No. of cases					0.394				0.586				0.454
≤200	2	5.22 (0.78, 35.09)	69.3	0.071		1.95 (1.36, 2.81)	23.2	0.254		1.33 (0.91, 1.95)	77.0	0.037	
>200	3	2.27 (1.78, 2.88)	0.0	0.773		1.74 (1.41, 2.15)	0.0	0.585		1.14 (1.00, 1.30)	90.1	<0.001	
DII components					0.355				0.729				0.782
<27	3	2.59 (1.86, 3.61)	43.2	0.172		1.85 (1.44, 2.37)	8.6	0.335		1.21 (0.99, 1.47)	87.7	<0.001	
≥27	2	2.16 (1.60, 2.93)	0.0	0.640		1.73 (1.32, 2.26)	0.0	0.549		1.17 (1.10, 1.24)	0.0	0.378	
E-DII					0.942				0.456				0.395
No	1	2.38 (1.52, 3.72)	–	–		1.54 (1.00, 2.38)	–	–		1.24 (1.10, 1.38)	–	–	
Yes	4	2.34 (1.80, 3.03)	31.6	0.226		1.85 (1.51, 2.26)	0.0	0.549		1.15 (1.03, 1.29)	86.4	<0.001	
Adjustment													
Age/gender					0.417				0.683				0.181
Yes	3	2.26 (1.74, 2.94)	0.0	0.763		1.82 (1.49, 2.24)	0.0	0.701		1.11 (1.00, 1.24)	87.2	<0.001	
No	2	5.08 (0.73, 35.33)	71.6	0.061		1.65 (1.08, 2.53)	44.1	0.181		1.38 (1.03, 1.84)	62.0	0.105	
SES					0.052				0.223				0.039
Yes	4	2.29 (1.83, 2.87)	0.0	0.902		1.77 (1.47, 2.13)	0.0	0.757		1.14 (1.03, 1.26)	86.7	<0.001	
No	1	18.46 (2.28, 149.72)	–	–		5.43 (0.90, 32.65)	–	–		1.69 (1.18, 2.43)	–	–	
BMI					0.545				0.545				0.301
Yes	2	2.22 (1.67, 2.96)	0.0	0.502		2.22 (1.67, 2.96)	0.0	0.502		1.11 (0.95, 1.29)	92.1	<0.001	
No	3	2.56 (1.79, 3.67)	43.5	0.170		2.56 (1.79, 3.67)	43.5	0.170		1.23 (1.08, 1.40)	60.4	0.080	
Family history of cancer					0.700				0.485				<0.001
Yes	1	2.57 (1.54, 4.29)	–	–		1.96 (1.43, 2.67)	–	–		1.03 (1.00, 1.06)	–	–	
No	4	2.30 (1.79, 2.95)	28.6	0.240		1.71 (1.36, 2.14)	0.0	0.536		1.19 (1.13, 1.26)	40.9	0.166	

DII, dietary inflammatory index; OR, odds ratio; CI, confidence interval; NOS, Newcastle–Ottawa Quality Assessment Scale; E-DII, energy adjusted DII; SES, Socio-economic status; BMI, body mass index.

a p-value for heterogeneity within each subgroup.

b p-value for heterogeneity between subgroups using Z-test.

## Discussion

To our knowledge, this is the first systematic review and dose–response meta-analysis investigating the association of DII score with risk of oral cancer. In our meta-analysis, the risk of oral cancer was increased by 135% at the highest DII level compared to the lowest DII level, and 79% at higher DII level compared to lower DII level. In addition, there was a positive linear dose–response association between DII score and oral cancer risk, with a 17% increased risk for a 1-unit increment in DII score. The overall results were consistent in most subgroup analyses. However, the risk of oral cancer appeared to be attenuated in the subgroup adjusted for SES or family history of cancer in the model.

In recent years, the associations between DII and site-specific cancers have been reported (14–17, 36). Hua et al. and Zhu et al. conducted two systematic reviews and dose–response meta-analyses on DII and upper aerodigestive tract cancer (UADT) (23, 24). Considering that oral cancer was a major subtype of UADT, our meta-analysis was, to some extent, comparable to the two prior meta-analyses. Hua et al. and Zhu et al. discovered that the pooled ORs of UADT cancer risk for the highest DII versus the lowest DII were 2.27 (1.89, 2.73) and 2.07 (1.82, 2.35), respectively (23, 24). The corresponding OR in our study was 2.35 (1.88, 2.94), which seemed to be similar to the ORs of the two prior studies. Likewise, the pooled OR of cancer for a 1-unit increment in DII score showed no significant difference between ours and Zhu et al.’s [1.17 (1.05, 1.30) vs. 1.18 (1.15, 1.21)] (24). However, the shapes of the curves representing the dose–response relationship between DII scores and cancer were somewhat different. To be specific, although there was a rising trend in both studies, our study showed a linear relationship rather than a nonlinear one as in the study by Zhu et al. (24). All of the above comparisons were not difficult to explain, probably because oral cancer has both common and unique features compared to UADT. Moreover, in comparison with the meta-analysis by Zhu et al., our meta-analysis included limited number of studies that might result in unstable results especially for the curve that represented the dose–response relationship.

In this meta-analysis, we conducted subgroup analyses to examine the heterogeneity between subgroups. Our results showed that association between DII and oral cancer risk was weaker within studies stratified to adjust for family history of cancer [1.03 (1.00, 1.06) vs. 1.19 (1.13, 1.26), *p* < 0.001]. This finding was not consistent with previous meta-analyses. For example, Wang et al. reported a stronger association between DII and breast cancer in studies stratified to adjust for family history (37). Guo et al. discovered no heterogeneity between subgroups by adjusting for family history of cancer (38). The inconsistent results may have contributed to the different types of cancer studied by these meta-analyses. Furthermore, only one study was adjusted for family history of cancer in our meta-analysis, which may lead to unstable or inaccurate results. We also discovered that the association between DII and oral cancer risk was influenced by adjustment for SES. That is to say, the effect size appeared to be larger within the subgroup not adjusting for SES. However, the result should be presented with caution, because only one study was included in the subgroup without adjustment for SES.

As known, the DII score was developed to reflect inflammation potentials of diet (11). Therefore, in the current review, we focused on inflammation to interpret the potential mechanism between DII score and oral cancer risk. In the DII scoring system, specific dietary components were associated with inflammation in different ways. For example, energy, carbohydrates, and total fats in diets contributed to body weight gain or elevated BMI, which might exacerbate the inflammatory status (39). As anti-inflammatory components in DII, selenium ameliorated inflammation by decreasing oxidative stress, and vitamin E by reducing proinflammatory cytokine expression (40, 41). In fact, inflammation has been indicated to be implicated in various types of cancer (42–44). The shared mechanisms may exist as follows. Certain inflammatory cytokines such as IL-1β, IL-6, and TNF-α may cause leukocyte infiltration, accumulation of macrophages, and activation of transcription factors, which, in turn, lead to inappropriate gene expression, cell proliferation, angiogenesis, and resistance to apoptosis (45–50). In addition, inflammation also played a critical role in the pathogenesis of several oral potentially malignant lesions, such as oral submucous fibrosis, oral lichen planus, and repetitive oral ulcers (51). Oral submucous fibrosis is mostly caused by long-term chewing of betel nut, which contains many specific chemical agents that induce the production of inflammatory mediators and growth factors including prostaglandins, TNF-α, IL-6, transforming growth factor-beta (TGF-β), and basic fibroblast growth factor. These biological mediators could drive the process of fibrosis through inducing downregulating collagenase production, upregulating collagen synthesis, and proliferation of fibroblasts (52–54). In addition, such a persistent inflammatory microenvironment of the submucous fibrosis may further favor malignant transformation to oral cancer (55). Oral lichen planus is generally considered to be a T cell-mediated mucocutaneous inflammatory disease with immune infiltration of CD8^+^ T cells and CD4^+^ T cells, and upregulated expression of inflammatory mediators such as COX-2, MMP-7, TNF-α, and IL-6 (56–58). Some researchers also proposed that such cytokines and chemokines that triggered oral lichen planus may further lead to the progression of oral lichen planus to oral squamous cell carcinoma by inducing fundamental changes of proteins in oral epithelial cells (59).

Our study has several strengths. Primarily, to the best of our knowledge, this is the first dose–response meta-analysis to quantify the relationship between DII score and oral cancer risk. Second, the risk of oral cancer with DII score was evaluated from various aspects, including ORs for the highest DII level versus the lowest, a higher DII level versus a lower one, 1-unit increment in DII score, and linear and non-linear dose–response relationships. Finally, we performed subgroup analyses by a considerable number of variables, and sensitivity analyses to discover the sources of heterogeneity.

There are also some limitations in this meta-analysis. First, all studies included in our meta-analysis were case–control design. Therefore, selection bias and recall bias were inevitable, which may lead to inaccurate results. Second, as the exposure variable, DII was not exactly the same in the included studies. Nevertheless, when we performed subgroup analyses based on number of components included in DII and whether it was E-DII, the heterogeneity between subgroups were not statistically significant. Third, this meta-analysis included only five studies, which warrants additional research in the future. However, our results may still have certain implications for the reason that the five studies involved most regions globally including Asia, Europe, North America, and South America. Finally, we discovered evidence of heterogeneity among studies when examining the risk of oral cancer at 1-unit increase in DII score. As a result, the random-effects model was applied to account for the heterogeneity, and the results should be reported with great caution.

Nowadays, along with the increasing global burden of oral cancer, it is gaining more and more attention, especially in relation to diet. In fact, oral cancer is one of the cancers most closely related to diet because food and nutrients are in direct contact with oral cavity. DII now provides an additional comprehensive approach in terms of diet to assessing oral cancer risk. It seems feasible to develop or modify dietary patterns based on DII in dietary guideline for residents. In oral cancer screening program, DII may also be applied as a component of screening for high-risk groups. In addition, a lower DII score may be beneficial in improving prognosis, which of course requires further study. In the future, DII may have broad and important application prospects in the prevention and control of oral cancer.

## Conclusion

In conclusion, the present meta-analysis suggested that a more pro-inflammatory diet, represented by the higher DII score, was associated with an elevated risk of oral cancer. Therefore, reducing pro-inflammatory food components and promoting anti-inflammatory food components would be beneficial in the prevention and control of oral cancer. In the future, additional high-quality studies are wanted to validate our results derived from limited research.

## Data availability statement

The original contributions presented in the study are included in the article/**Supplementary Material**. Further inquiries can be directed to the corresponding authors.

## Author contributions

ZL conceived, designed, and performed the work; ZL analyzed the data; XZ, YH, SY and LC revised the manuscript. All authors gave final approval and agree to be accountable for all aspects of work ensuring integrity and accuracy.

## Funding

This study was supported by the Science and Technology Innovation Program of Hunan Province (grant no. 2022SK2050) and the National Natural Science Foundation Program of China (grant nos. 81973137 and 82173608).

## Acknowledgments

We would like to thank all the authors of the included studies.

## Conflict of interest

The authors declare that the research was conducted in the absence of any commercial or financial relationships that could be construed as a potential conflict of interest.

## Publisher’s note

All claims expressed in this article are solely those of the authors and do not necessarily represent those of their affiliated organizations, or those of the publisher, the editors and the reviewers. Any product that may be evaluated in this article, or claim that may be made by its manufacturer, is not guaranteed or endorsed by the publisher.
