# Supplementary material for: Association between dietary inflammatory index and oral cancer risk: A systematic review and dose–response meta-analysis
Source: Front Oncol. 2022 Sep 26;12:920452. doi: 10.3389/fonc.2022.920452 (PMC9548600; doi:10.3389/fonc.2022.920452)
Supplement: Supplementary file 1 [file DataSheet_1.docx]

Supplementary Table 1. Systematic literature review search terms and strategy.

| **Literature search strategy in PubMed** |
| --- |
| 1: dietary inflammatory index OR dietary inflammatory score OR dietary score OR inflammatory diet OR inflammatory potential of diet OR dietary inflammation potential OR inflammatory potential intake OR anti-inflammatory diet OR pro-inflammatory diet OR dietary pattern OR diet-related inflammation OR index-based dietary patterns OR DII |
| 2: mouth neoplasms [MeSH Terms] OR mouth neoplasms OR mouth neoplasm OR oral neoplasms OR oral neoplasm OR mouth cancers OR mouth cancer OR oral cancers OR oral cancer OR cancer of mouth OR cancer of the mouth OR oral squamous cell carcinoma OR oral squamous cell cancer |
| 3: 1 AND 2 |
| **Literature search strategy in Cochrane Library** |
| 1: (dietary inflammatory index) OR (dietary inflammatory score) OR (dietary score) OR (inflammatory diet) OR (inflammatory potential of diet) OR (dietary inflammation potential) OR (inflammatory potential intake) OR (anti-inflammatory diet) OR (pro-inflammatory diet) OR (dietary pattern) OR (diet-related inflammation) OR (index-based dietary patterns) OR (DII) |
| 2: (MeSH descriptor: [Mouth Neoplasms] explode all trees) OR (mouth neoplasms) OR (mouth neoplasm) OR (oral neoplasms) OR (oral neoplasm) OR (mouth cancers) OR (mouth cancer) OR (oral cancers) OR (oral cancer) OR (cancer of mouth) OR (cancer of the mouth) OR (oral squamous cell carcinoma) OR (oral squamous cell cancer) |
| 3: 1 AND 2 |
| **Literature search strategy in EMBASE** |
| 1: ‘dietary inflammatory index’ OR ‘dietary inflammatory score’ OR ‘dietary score’ OR ‘inflammatory diet’ OR ‘inflammatory potential of diet’ OR ‘dietary inflammation potential’ OR ‘inflammatory potential intake’ OR ‘anti-inflammatory diet’ OR ‘pro-inflammatory diet’ OR ‘dietary pattern’ OR ‘diet-related inflammation’ OR ‘index-based dietary patterns’ OR ‘DII’ |
| 2: ‘mouth neoplasms’/exp OR ‘mouth neoplasms’ OR ‘mouth neoplasm’ OR ‘oral neoplasms’ OR ‘oral neoplasm’ OR ‘mouth cancers’ OR ‘mouth cancer’ OR ‘oral cancers’ OR ‘oral cancer’ OR ‘cancer of mouth’ OR ‘cancer of the mouth’ OR ‘oral squamous cell carcinoma’ OR ‘oral squamous cell cancer’ |
| 3: 1 AND 2 |
| **Literature search strategy in Web of Science** |
| 1: Search TS = (dietary inflammatory index OR dietary inflammatory score OR dietary score OR inflammatory diet OR inflammatory potential of diet OR dietary inflammation potential OR inflammatory potential intake OR anti-inflammatory diet OR pro-inflammatory diet OR dietary pattern OR diet-related inflammation OR index-based dietary patterns OR DII) |
| 2: Search TS = (mouth neoplasms OR mouth neoplasm OR oral neoplasms OR oral neoplasm OR mouth cancers OR mouth cancer OR oral cancers OR oral cancer OR cancer of mouth OR cancer of the mouth OR oral squamous cell carcinoma OR oral squamous cell cancer) |
| 3: 1 AND 2 |

Supplementary Table 2. Food parameters included in DII and covariates adjusted in the model in studies included.

| Author, year, country | DII components | Covariates adjusted in the model |
| --- | --- | --- |
| Bao et al., 2020, China [18] | 22 parameters:  energy, fiber, carbohydrate, protein, cholesterol, carotene, total fat, MUFA, PUFA, SFA, niacin, vitamin A, vitamin B_12_, vitamin B_2_, vitamin B_1_, vitamin C, vitamin E, folate, selenium, zinc, iron, magnesium. | Age, gender, occupation, education level, marital status, residence, BMI, tobacco smoking, alcohol drinking, tea consumption, family history of cancer. |
| Secchi et al., 2019, Argentina [19] | 25 parameters:  carbohydrates, proteins, fat, energy, cholesterol, iron, vitamin A, riboflavin, vitamin B_6_, vitamin C, vitamin E, selenium, zinc, ethanol, fiber, tea, omega-3, omega-6, saturated fat, garlic, onion, MUFA, PUFA, caffeine, omega-6/omega-3 ratio. | Alcohol and tobacco consumption. |
| Mazul et al., 2018, America [20] | 27 parameters:  carbohydrate, protein, total fat, alcohol, fiber, cholesterol, saturated fat, mono-unsaturated fat, poly-unsaturated fat, omega-3, omega-6, trans-fat, niacin, thiamin, riboflavin, vitamin A, vitamin B_12_, vitamin B_6_, vitamin C, vitamin D, vitamin E, iron, magnesium, zinc, selenium, folic acid, beta carotene. | Education, income, smoking, total lifetime alcohol intake, age, race, and sex. |
| Abe et al., 2018, [Japan](D:/%E5%AE%89%E8%A3%85%E5%8C%85/%E6%9C%89%E9%81%93%E7%BF%BB%E8%AF%91/Youdao/Dict/8.5.3.0/resultui/html/index.html#/javascript:;) [21] | 22 parameters:  energy, protein, fat, carbohydrate, total dietary fiber, cholesterol, SFA, MUFA, PUFA, omega-3, omega-6, carotene, vitamin B_1_, vitamin B_2_, vitamin B_6_, vitamin B_12_, vitamin C, vitamin D, vitamin E, retinoic acid, folate, iron. | Smoking, ethanol consumption, flushing phenotype, teeth and occupation group. |
| Shivappa et al., 2017, Italy [22] | 31 parameters:  Carbohydrates, proteins, fats, fiber, cholesterol, SFA, MUFA, PUFA, omega-3, omega-6, niacin, thiamin, riboflavin, vitamin B_6_, iron, zinc, vitamin A, vitamin C, vitamin D, vitamin E, folic acid, b-carotene, anthocyanidins, flavan-3-ols, flavonols, flavanones, flavones, isoflavones, caffeine, alcohol, tea. | Age, sex, center, year of interview, and non-alcohol energy intake, and additionally adjusted for education, tobacco smoking, alcohol drinking, and BMI. |

Abbreviations: DII, dietary inflammatory index; MUFA, monounsaturated fatty acid; PUFA, polyunsaturated fatty acid; omega-3, omega-3 fatty acid; omega-6, omega-6 fatty acid; SFA, saturated fatty acid; BMI, body mass index.

| Items | Standards | Bao et al. 2019 [18] | Secchi et al. 2019 [19] | Mazul et al. 2018 [20] | Abe et al. 2018 [21] | Shivappa et al. 2017 [22] |
| --- | --- | --- | --- | --- | --- | --- |
| 1. Is the case definition adequate? | 1.Yes, with independent validation* | 1 | 1 | 1 | 1 | 1 |
|  | 2.Yes, eg record linkage or based on self reports |  |  |  |  |  |
|  | 3.No description |  |  |  |  |  |
| 2. Representativeness of the cases | 1.Consecutive or obviously representative series of cases* | 1 | 1 | 1 | 1 | 1 |
|  | 2.Potential for selection biases or not stated |  |  |  |  |  |
| 3. Selection of controls | 1.Community controls* |  |  | 1 |  |  |
|  | 2.Hospital controls | 0 | 0 |  | 0 | 0 |
|  | 3.No description |  |  |  |  |  |
| 4. Definition of controls | 1.No history of disease (endpoint)* | 1 | 1 | 1 | 1 | 1 |
|  | 2.No description of source |  |  |  |  |  |
| Comparability of cases and controls on the basis of the design or analysis | 1.Study controls for the most important factor* | 1 | 1 | 1 | 1 | 1 |
|  | 2.Study controls for any additional factor (This criteria could be modified to indicate specific control for a second important factor)* | 1 | 1 | 1 | 1 | 1 |
| 1. Ascertainment of exposure | 1.Secure record (eg surgical records)* |  |  |  |  |  |
|  | 2.Structured interview where blind to case/control status* |  |  |  |  |  |
|  | 3.Interview not blinded to case/control status |  | 0 |  |  | 0 |
|  | 4.Written self report or medical record only |  |  |  | 0 |  |
|  | 5.No description | 0 |  | 0 |  |  |
| 2. Same method of ascertainment for cases and controls | 1.Yes* | 1 | 1 | 1 | 1 | 1 |
|  | 2.No |  |  |  |  |  |
| 3. Non-Response Rate | 1.Same rate for both groups* |  |  |  |  | 1 |
|  | 2.Non respondents described | 0 | 0 |  | 0 |  |
|  | 3.Rate different and no designation |  |  | 0 |  |  |
|  | **Total score** | 6 | 6 | 7 | 6 | 7 |

Supplementary Table 3. Newcastle-Ottawa Quality Assessment Scale (NOS) score of included studies.


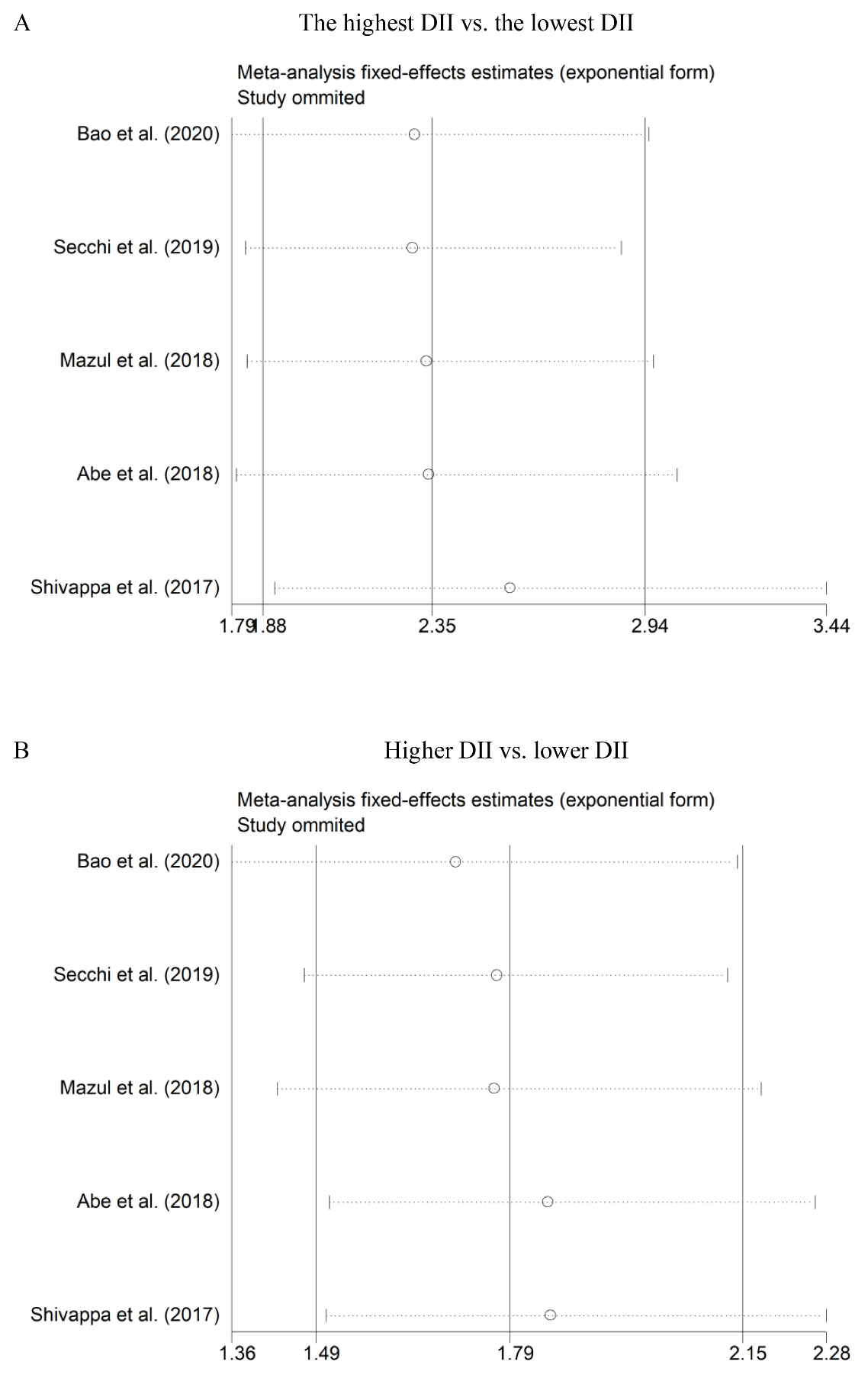


**Supplementary Figure 1.** Sensitivity analysis on the association between DII and oral cancer risk. (A) The highest DII versus the lowest DII. (B) Higher DII versus lower DII.

Notes: DII, dietary inflammatory index.
